# Apabetalone (RVX-208) reduces vascular inflammation in vitro and in CVD patients by a BET-dependent epigenetic mechanism

**DOI:** 10.1186/s13148-019-0696-z

**Published:** 2019-07-12

**Authors:** Laura M. Tsujikawa, Li Fu, Shovon Das, Christopher Halliday, Brooke D. Rakai, Stephanie C. Stotz, Christopher D. Sarsons, Dean Gilham, Emily Daze, Sylwia Wasiak, Deborah Studer, Kristina D. Rinker, Michael Sweeney, Jan O. Johansson, Norman C. W. Wong, Ewelina Kulikowski

**Affiliations:** 1 Resverlogix Corp., 300, 4820 Richard Road SW, Calgary, AB T3E 6 L1 Canada; 2 Cellular and Molecular Bioengineering Research Lab, Libin Cardiovascular Institute of Alberta, University of Calgary, HMRB 358/361 3330 University Drive NW, Calgary, AB T2N 4 N1 Canada; 3 Resverlogix Inc., Suite 4010, 44 Montgomery Street, San Francisco, CA 94104 USA

**Keywords:** Apabetalone, Bromodomain, BRD4, Epigenetics, THP-1 monocytes, HUVEC, endothelium, Adhesion, Atherosclerosis, CVD, Diabetes, Vascular inflammation

## Abstract

**Background:**

Apabetalone (RVX-208) is a bromodomain and extraterminal protein inhibitor (BETi) that in phase II trials reduced the relative risk (RR) of major adverse cardiac events (MACE) in patients with cardiovascular disease (CVD) by 44% and in diabetic CVD patients by 57% on top of statins. A phase III trial, BETonMACE, is currently assessing apabetalone’s ability to reduce MACE in statin-treated post-acute coronary syndrome type 2 diabetic CVD patients with low high-density lipoprotein C. The leading cause of MACE is atherosclerosis, driven by dysfunctional lipid metabolism and chronic vascular inflammation (VI). In vitro studies have implicated the BET protein BRD4 as an epigenetic driver of inflammation and atherogenesis, suggesting that BETi may be clinically effective in combating VI. Here, we assessed apabetalone’s ability to regulate inflammation-driven gene expression and cell adhesion in vitro and investigated the mechanism by which apabetalone suppresses expression. The clinical impact of apabetalone on mediators of VI was assessed with proteomic analysis of phase II CVD patient plasma.

**Results:**

In vitro, apabetalone prevented inflammatory (TNFα, LPS, or IL-1β) induction of key factors that drive endothelial activation, monocyte recruitment, adhesion, and plaque destabilization. BRD4 abundance on inflammatory and adhesion gene promoters and enhancers was reduced by apabetalone. BRD2-4 degradation by MZ-1 also prevented TNFα-induced transcription of monocyte and endothelial cell adhesion molecules and inflammatory mediators, confirming BET-dependent regulation. Transcriptional regulation by apabetalone translated into a reduction in monocyte adhesion to an endothelial monolayer. In a phase II trial, apabetalone treatment reduced the abundance of multiple VI mediators in the plasma of CVD patients (SOMAscan® 1.3 k). These proteins correlate with CVD risk and include adhesion molecules, cytokines, and metalloproteinases. Ingenuity® Pathway Analysis (IPA®) predicted that apabetalone inhibits pro-atherogenic regulators and pathways and prevents disease states arising from leukocyte recruitment.

**Conclusions:**

Apabetalone suppressed gene expression of VI mediators in monocytes and endothelial cells by inhibiting BET-dependent transcription induced by multiple inflammatory stimuli. In CVD patients, apabetalone treatment reduced circulating levels of VI mediators, an outcome conducive with atherosclerotic plaque stabilization and MACE reduction. Inhibition of inflammatory and adhesion molecule gene expression by apabetalone is predicted to contribute to MACE reduction in the phase III BETonMACE trial.

**Electronic supplementary material:**

The online version of this article (10.1186/s13148-019-0696-z) contains supplementary material, which is available to authorized users.

## Introduction

**Fig. 1:**
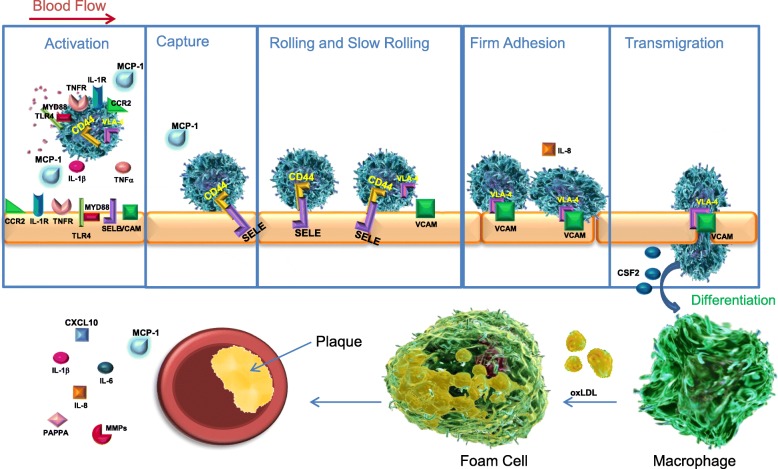
The multi-step process of atherogenesis: Activation of the endothelium, monocyte activation and recruitment, leukocyte capture, rolling, adhesion, firm adhesion, macrophage differentiation, plaque development and stability. Apabetalone downregulates the transcription of each protein labeled in the illustration, thus impacting each step of atherogenesis. At the plaque, circulating and local cytokine expression from endothelial cells and monocytes are downregulated by apabetalone. Activation panel: pink speckles represent multiple cytokine secretion

Vascular inflammation (VI) is a driver of atherosclerosis and is exacerbated by hypertension, hypercholesterolemia, and diabetes mellitus [1]. Sustained high levels of circulating cytokines activate the endothelium and circulating monocytes, leading to an increase in cellular adhesion and further cytokine production. Monocytes are recruited to the activated endothelium where they adhere and transmigrate through the arterial walls (Fig. 1). Following transendothelial migration, monocytes differentiate into macrophages, taking up oxidized low-density lipoprotein (oxLDL) and forming foam cells, which leads to atherosclerotic plaque formation [2, 3]. Plaque stability itself is complex, with many contributors: infiltrating immune cells, plaque lipid and collagen content, and extracellular matrix remodeling factors [4]. If the plaque becomes unstable and ruptures, thrombosis, stroke, or myocardial infarction (MI) can occur. One in three global deaths are estimated to be a consequence of cardiovascular disease (CVD)-related events such as these [5]. Despite numerous pharmacological interventions, this incredible healthcare burden remains. New therapeutic strategies are needed to address the residual unmet need.

**Fig. 2:**
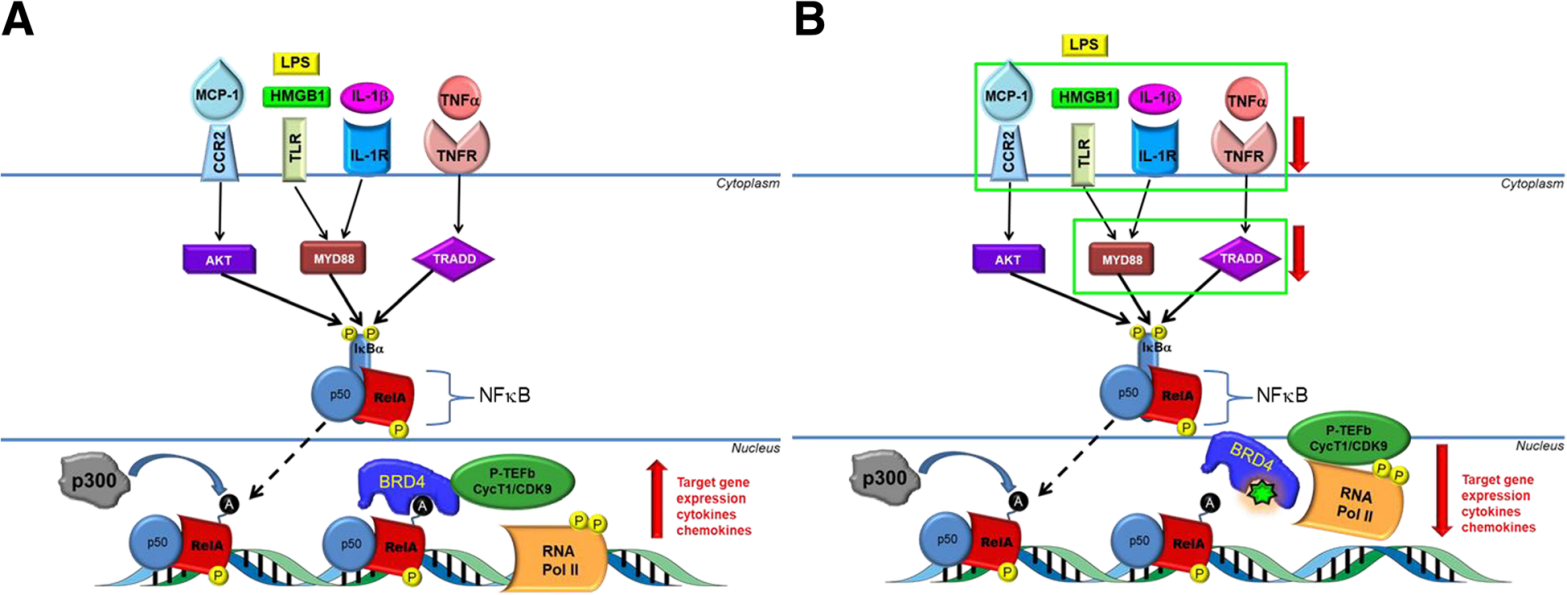
Convergent inflammatory signaling through NF-κB potentiates BRD4-dependent transcription of VI mediators, a result suppressed by apabetalone. **a** MCP-1, LPS, IL-1β, and TNFα all signal through NF-κB. The stimulants MCP-1, LPS, IL-1β, and TNFα activate their cognate receptors CCR2, TLR, IL-1R, and TNFR respectively. The receptors translate the signal through AKT, MYD88 and TRADD, phosphorylating NF-κB (yellow “p” circles) and releasing RelA-p50 subunits from IκBα. RelA translocates to the nucleus where it binds to consensus DNA binding sequences and is acetylated at K310 by p300 (black “a” circles). BRD4 recognizes and binds to these acetylation marks, recruiting pTEFb to activate RNA Pol II to drive inflammatory gene expression (cytokines, chemokines, and adhesion molecules). **b** Apabetalone (green 7-point star) competitively inhibits BRD4 BD2 interactions with acetylated lysine marks on RelA. This prevents pTEFb recruitment and Pol II activation, inhibiting the transcription of VI mediators and components of the NF-κB pathway. Green boxes and red arrows indicate genes in the illustration whose expression is reduced by apabetalone

In cell nuclei, bromodomain and extraterminal (BET) proteins bind to acetylated histones and transcription factors and act as molecular scaffolds between chromatin and transcriptional machinery to regulate gene expression [6–9]. Under basal conditions, BET protein occupancy at enhancer and promoter sites on chromatin support expression of housekeeping genes important for cell maintenance and metabolism. In response to inflammatory stimuli, signaling via tumor necrosis factor receptor (TNFR), toll-like receptor (TLR), interleukin 1 receptor (IL-1R), and their respective ligands induces the translocation of the master inflammatory transcription factor nuclear factor kappa-light-chain-enhancer of activated B cells (NF-κB) from the cytoplasm to the nucleus (Fig. 2a) [10, 11]. Nuclear NF-κB binds to DNA *cis*-regulatory response elements at enhancers and promoters, where the NF-κB RelA/p65 subunit is acetylated [12]. RelA acetylation recruits BRD4 from housekeeping genes to pro-inflammatory genes, forming de novo super-enhancer (SE) sites to which positive transcription elongation factor complex (P-TEFb) and chromatin remodeling factors are also recruited [6]. Consequently, NF-κB target genes are transcribed, driving inflammation. Sustained NF-κB signaling leads to pathologies such as atherosclerosis, hypertrophy, and hypertension [13, 14].

BET inhibitors (BETi) are small molecule epigenetic modifiers with great therapeutic potential. BETi bind to the bromodomains (BD1 and BD2) of BET proteins (BRD2, BRD3, BRD4, and BRDT), preventing their interaction with acetylated lysines on histone tails and transcription factors [15–17]. Pan-BETi bind to BD1 and BD2 equally, while selective BETi have a greater affinity for one BD over the other. The pan-BETi JQ1 prevents inflammation-induced redistribution of BRD4 on the chromatin and suppresses tumor necrosis factor alpha (TNFα)-induced expression of inflammatory and adhesion molecules in endothelial cells [13, 14]. JQ1 also significantly reduces monocyte (THP-1 cell) adhesion to human umbilical vein endothelial cells (HUVECs) and inhibits atherogenesis in hypercholesterolemic mice [13]. These findings highlight BETi as potential anti-inflammatory agents in atherosclerosis. Pan-BETi cannot be administered chronically due to toxic effects [18], and BD1-specific BETi are currently untested in humans. Therefore, it is imperative to determine whether a BD2-selective BETi maintains anti-inflammatory properties without toxicity.

Apabetalone (RVX-208) is an oral small molecule inhibitor of bromodomain and extraterminal (BET) proteins with BD2 selectivity [19, 20]. It is extremely well tolerated by patients, with safety data now exceeding 2700 patient years. The only dose-limiting toxicity observed to date is a reversible and transient elevation in alanine aminotransferases/aspartate aminotransferases in a small percentage of patients [21]. This is managed by periodic measurement of hepatic transaminases with discontinuation of the drug if levels greater than five times the upper limit of normal are observed. This occurs in < 4% of patients in clinical trials. Further, apabetalone significantly reduced both plaque size and vulnerability (ASSURE trial) [22]. In vitro, apabetalone has been shown to reduce pro-inflammatory gene expression in human aortic endothelial cells (HAEC) and macrophage-like U937 cells [23]. In vivo, prophylactic and therapeutic apabetalone treatment significantly reduced aortic lesion formation and lowered levels of circulating adhesion molecules and cytokines in hyperlipidemic ApoE (−/−) mice [23]. These findings suggest that apabetalone combats VI and has potential to reduce atherogenesis in CVD patients.

Here, we demonstrate that BET proteins are necessary for the expression of genes that drive VI and atherosclerosis. We reveal that the BD2-selective BETi, apabetalone, prevents inflammation-induced BRD4 accumulation on the enhancers and promoters of VI genes in endothelial cells. Transcription of genes involved in inflammation, endothelial dysfunction, monocyte recruitment, and plaque instability are subsequently suppressed by apabetalone treatment. This reduces the abundance of critical adhesion and monocyte recruitment proteins and functionally inhibits monocyte adhesion to inflamed endothelial cells under both static and flow conditions. We further report that apabetalone reduces the abundance of VI mediators in the plasma of CVD patients treated for 6 months (phase II trial). Our data provide a mechanism by which BD2-selective apabetalone suppresses VI through BET-dependent epigenetic transcriptional regulation. This regulation translates into a reduction in VI mediators in CVD patient plasma, which correlates with an observed reduction in plaque parameters and major adverse cardiac events (MACE).

## Results

### Apabetalone inhibits BRD4 interactions with chromatin at regulatory sites in endothelial cells

**Fig. 3:**
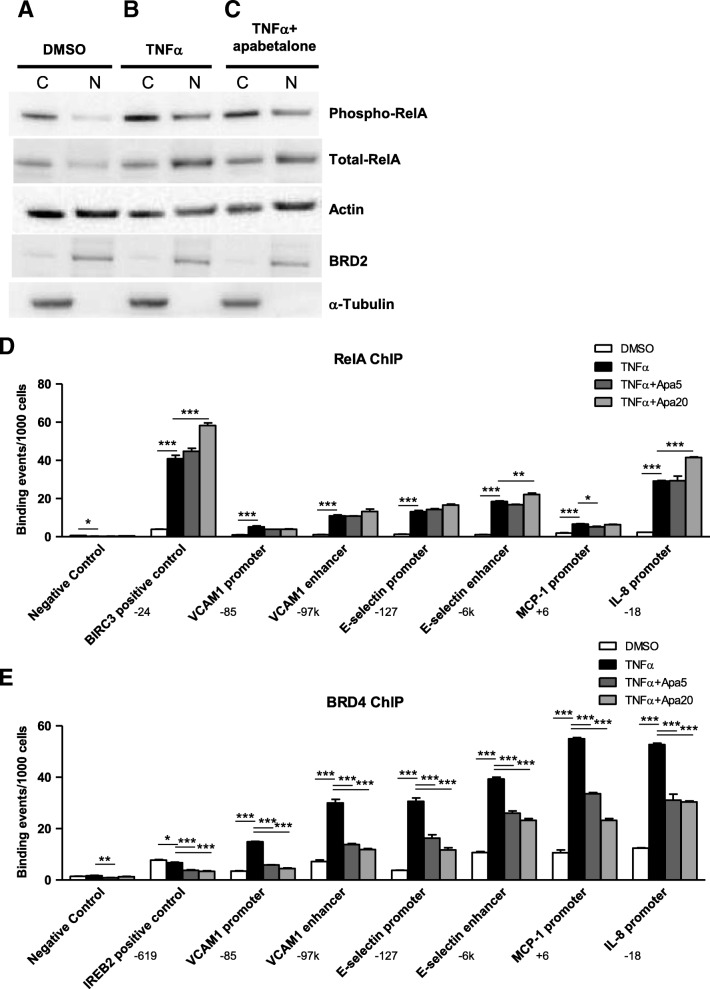
Apabetalone does not interfere with NF-kB translocation from the cytoplasm to the nucleus or association of RelA with the chromatin shown via western blot and ChIP. **a** Western blot: Phospho-RelA and total-RelA is found almost exclusively in the HUVEC cytoplasm (C) under unstimulated conditions (DMSO). **b** TNFα stimulation induces phospho-RelA and total-RelA translocation to the nucleus (N). **c** Apabetalone (20 μM) co-treatment (2 h) does not alter translocation. **a**–**c** The loading control used was β-actin, the nuclear protein control was BRD2 and cytoplasmic control was α-tubulin. **d** ChIP: RelA occupancy on the VCAM1 enhancer and promoter, the SELE enhancer and promoter, and the promoters of MCP-1 and IL-8 increases substantially with TNFα stimulation. Apabetalone (5 and 20 μM) does not reduce RelA occupancy. **e** BRD4 occupancy on the VCAM1 enhancer and promoter, the SELE enhancer and promoter, and the promoters of MCP-1 and IL-8 also increases substantially with TNFα stimulation. Apabetalone (5 and 20 μM) diminishes BRD4 occupancy at each of these sites. ChIP locations from transcriptional start sites are indicated by the target gene. Statistical significance was determined through 1-way ANOVA analysis followed by Dunnett’s Multiple Comparison Test using TNFα response for the comparison, where *p < 0.05, **p < 0.01, ***p < 0.001

The impact of apabetalone on RelA/p65 translocation and BRD4 chromatin occupancy was assessed to determine if the BD2-specific apabetalone suppresses VI transcription through the same mechanism as the pan-BETi JQ1 [13]. In response to TNFα stimulation, phosphorylated RelA/p65 translocated from the cytoplasm to the nucleus (Fig. 3a, b) to initiate the expression of pro-inflammatory genes. Apabetalone treatment did not reduce RelA/p65 translocation (Fig. 3c); comparable levels of phospho-RelA/p65 and total RelA/p65 were measured in the nucleus of HUVECs following TNFα treatment with and without apabetalone. Further, apabetalone did not alter TNFα-induced redistribution of RelA/p65 to the enhancers and promoters of adhesion molecules or inflammatory mediators (ChIP; Fig. 3d). TNFα also increased the abundance of BRD4 bound to adhesion molecule enhancers and promoters and cytokine promoters (Fig. 3e). In contrast, apabetalone treatment suppressed the redistribution of BRD4 to these sites. Apabetalone did not alter the overall abundance of BRD4 (Additional file 1: Figure S1). These data show that the BD2-selective BETi apabetalone decreases BRD4 chromatin occupancy at regulatory sites of pro-inflammatory genes similar to the pan-BETi JQ1 [13]. Moreover, gene transcription levels rapidly paralleled changes in BRD4 chromatin occupancy (Additional file 1: Figure S2); adhesion molecule and cytokine expression increased with TNFα treatment, and apabetalone pretreatment reduced induction to a similar extent as it reduced BRD4 chromatin occupancy.

### Induction of endothelial inflammation and adhesion mediators requires BET proteins

**Fig. 4:**
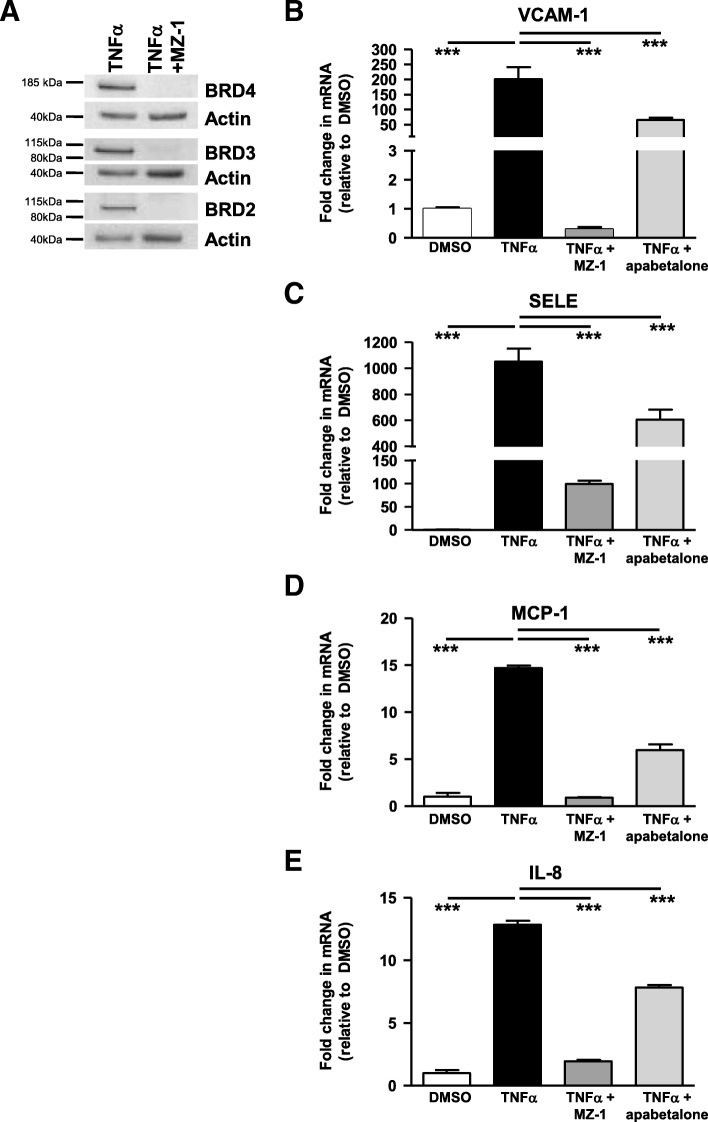
In endothelial cells, MZ-1 and apabetalone prevent TNFα induction of key inflammatory and adhesion marker transcripts through the degradation or inhibition of BET proteins respectively. **a** MZ-1 (1 μM; 6 h) degrades HUVEC BRD2, BRD3, and BRD4 as shown by western blot. **b**–**e** TNFα stimulation (2 h) fails to induce HUVEC transcription of *VCAM-1* (**b**), *MCP-1* (**d**), and *IL-8* (**e**) following MZ-1 pretreatment (4 + 2 h). *SELE* (**c**) induction is reduced but not eliminated. Apabetalone (20 μM) pretreatment (4 + 2 h) also decreases the level of inductions. Statistical significance was determined through 1-way ANOVA analysis followed by Dunnett’s Multiple Comparison Test using TNFα response for the comparison, where *p < 0.05, **p < 0.01, ***p < 0.001

To further demonstrate that BET proteins are necessary for VI gene expression changes, we knocked down BRD2-4 with MZ-1 in endothelial cells and assessed transcript levels after stimulus treatment (Fig. 4a). MZ-1 is a proteolysis targeting chimeric molecule (PROTAC), resulting from the fusion of the pan-BETi JQ1 and the ligand of E3 ubiquitin ligase VHL, designed to target BET proteins for ubiquitination and degradation [24]. After confirming BRD2-4 degradation by MZ-1, we measured gene expression changes following TNFα stimulation (Fig. 4b–e). TNFα induction of endothelial adhesion molecule (Fig. 4b, c ) and cytokine (Fig. 4d, e) gene expression was largely prevented by BET protein degradation. Apabetalone pretreatment also inhibited TNFα induction of these same genes (Fig. 4b–e), albeit to a lesser extent than with MZ-1. This was expected, as non-covalent small molecule inhibitory interactions are transient in nature. These data indicate that BET proteins are required for TNFα-induced expression of key VI genes and that BD2-selective BET inhibition by apabetalone can suppress the majority of this effect.

### Apabetalone represses cytokine and endotoxin induction of multiple VI mediator transcripts in endothelial cells

Widespread inflammatory responses that converge on NF-κB signaling (Fig. 2a) are key drivers of VI. To examine if apabetalone can disrupt these responses, we treated endothelial cells with TNFα, interleukin 1 beta (IL-1β), or lipopolysaccharide (LPS) and assessed gene expression (Table 1). The stimulants differentially induced HUVEC transcription of multiple cytokines, chemokines, TLR signaling molecules, and adhesion molecules. For instance, TNFα robustly induced E-selectin (*SELE*) over 1000 fold, while IL-1β increased expression ~ 370 fold and LPS 11 fold. Regardless of the extent of induction, however, apabetalone treatment suppressed the effects of each stimulant tested (Table 1), highlighting the central role BET proteins play in endothelial cell responses to inflammatory stimuli that activate NF-κB signaling.

**Table 1:** Apabetalone suppresses stimulant-induced transcripts of HUVEC cytokine, chemokine, TLR signaling, and adhesion molecules

Function	Gene	TNFα			IL-1β			LPS		
		Fold induction	% reduction		Fold induction	% reduction		Fold induction	% reduction	
		Control	Apabetalone		Control	Apabetalone		Control	Apabetalone	
			5 μM	20 μM		5 μM	20 μM		5 μM	20 μM
Enzyme	COX-2	4	NS	− 86	19	− 46	− 85	NI	− 42	− 83
Cytokine	CSF-2	945	− 82	− 98	8096	− 59	− 91	9	− 64	− 85
	IL-1β	1685	− 90	− 99	ND	ND	ND	ND	ND	ND
	IL-6	9	− 51	− 91	191	− 54	− 84	1.6	− 67	− 69
	IL-8	26	ND	− 48	ND	ND	ND	ND	ND	ND
	OPG	43	− 95	− 99	142	− 96	− 99	1.4	− 71	− 84
Chemokine	MCP-1	40	− 21	− 71	44	− 35	− 62	4	− 50	− 82
TLR signaling	MYD88	NI	NS	− 56	NI	− 30	− 66	1.6	− 44	− 38
Adhesion molecules	CD44	2	NS	− 34	3	NS	NS	NI	− 33	− 34
	SELE	1164	NS	− 54	368	− 17	− 40	11	− 51	− 76
	VCAM-1	196	− 59	− 83	96	− 72	− 91	6	− 73	− 96

NS no signal, NI no induction, ND not determined

To obtain a broader endothelial gene expression data set for bioinformatic analysis, we used the NanoString nCounter® Inflammation V2 gene expression panel (NanoString Technologies) to simultaneously assay expression of 255 genes. TNFα induced 109 genes by more than 1.3 fold (data not shown). Apabetalone suppressed the induction of 79 of those 109 transcripts, including chemokines, cytokines, transcription factors, receptors, and enzymes. Differences in absolute induction and reduction between data sets were the result of differences in assay sensitivities (Tables 1 and 2). The overlap in regulated genes, however, was extensive. Ingenuity® Pathway Analysis (IPA®) was used to predict endothelial upstream regulators, canonical pathways, and diseases and biological functions increased by TNFα (positive activation z-score> ~ 2) and inhibited by apabetalone treatment (negative activation z-score < ~ − 2). Predicted upstream regulators included the endotoxin LPS, cytokines, and TLR signaling molecules (Table 3). IPA® also predicted inhibition of multiple pro-inflammatory canonical pathways, including interleukin 6 (IL-6), NF-κB, and high mobility group box 1 (HMGB1) signaling, by apabetalone (Table 4). IPA® diseases and biological functions analysis highlighted processes involved in immune cell activation, movement, and recruitment as potentially counteracted by apabetalone (Table 5). Many of the endothelial genes repressed by apabetalone are associated with atherogenesis [25, 26]; their suppression is predicted to suppress atherosclerotic progression.

**Table 2:** HUVEC NanoString: apabetalone reduces the expression of pro-atherogenic genes upregulated by TNFα

Function	Gene	TNFα		
		Fold induction	% reduction	
		Control	Apabetalone	
			5 μM	20 μM
Chemokine	MCP-1^p^	133	− 18	− 65
	CXCL10^p^	86	− 78	− 84
	CXCL3	820	11	− 32
	IL-8^p^	185	− 47	− 79
Cytokine	CSF-2	154.6	− 76	− 93
	IL-1β^p^	3.0	− 64	− 66
	IL-15^p^	31.6	− 59	− 87
	IL-18^p^	8.4	65	− 25
	IL-6^p^	9.6	− 59	− 90
	LTB^p^	119.3	− 77	− 99
	TGFB3	23.5	− 30	− 88
	TNF	14.6	− 93	− 93
Transcription factor	IRF1^p^	7.9	− 26	− 42
	RELB	14.4	− 15	− 30
Receptor and Adaptors	TLR2^p^	28.9	− 52	− 78
	IL-1R	3.2	− 47	− 72
	MYD88	1.3	− 3	− 41
	TRADD	2.4	10	− 35
Enzymes	C1S^p^	11.1	− 90	− 91
	CFB^p^	72.6	− 64	− 87
	COX-2	8.5	− 59	− 93
	IFIT2^p^	11.1	− 23	− 15
	TNFAIP3	80.5	− 28	− 51

^P^Genes involved in plaque stability

**Table 3:** HUVEC Nanostring: IPA® upstream regulator analysis predicted that endotoxin, cytokine, and TLR signaling regulators are inhibited by apabetalone

IPA upstream regulators				
Ingenuity® Pathway Analysis	Target	z-score		
		TNFα	Apabetalone 5 μM	Apabetalone 20 μM
Endotoxin	LPS	6.3	− 3.3	− 4.4
Cytokines	TNFα	6.1	− 2.6	− 4.2
	NF-κB (complex)	5.9	− 2.7	− 3.5
	IL-1β	5.4	− 1.9	− 3.3
	IL-1α	4.9	− 2.2	− 1.8
	RELA	4.8	− 1.9	− 2.7
	IFNG	4.3	− 2.9	− 4.0
	IL-6	2.6	− 1.6	− 1.5
TLR signaling	TLR4	5.3	− 3.3	− 4.4
	TICAM1	5.1	− 2.7	− 4.5
	MYD88	5.1	− 3.1	− 4.1
	TLR3	5.0	− 3.3	− 3.5

Positive activation z-scores reflect the predicted activation of an upstream regulator (significant when > ~ 2)

Negative activation z-scores reflect the predicted inactivation of an upstream regulator (significant when < ~− 2)

**Table 4:** HUVEC Nanostring: IPA® canonical pathway analysis identified TNFα-activated pathways inhibited by apabetalone

IPA canonical pathways			
Ingenuity® Pathway Analysis	z-score		
	TNFα	Apabetalone 5 μM	Apabetalone 20 μM
TREM1 signaling	3.8	− 1.8	− 3.1
Acute phase response signaling	3.8	− 1.6	− 3.1
SAPK/JNK signaling	3.7	− 1.3	− 2.8
IL-6 signaling	3.7	− 0.8	− 2.2
Dendritic cell maturation	3.7	− 2.2	− 4.0
HGF signaling	3.6	− 0.5	− 2.1
Neuroinflammation signaling pathway	3.6	− 1.6	− 2.1
Renin-angiotensin signaling	3.5	0.4	− 2.1
HMGB1 signaling	3.4	− 1.2	− 2.2
NF-κB signaling	3.4	− 1.4	− 2.9

Positive activation z-scores reflect the predicted activation of a canonical pathway (significant when > ~ 2)

Negative activation z-scores reflect the predicted inactivation of a canonical pathway (significant when < ~− 2)

**Table 5:** HUVEC Nanostring: IPA® diseases and biological functions identified TNFα-activated processes inhibited by apabetalone

Diseases and biological functions			
Ingenuity® Pathway Analysis	z-score		
	TNFα	Apabetalone 5 μM	Apabetalone 20 μM
Migration of cells	6.0	− 1.4	− 4.1
Cell movement	5.9	− 1.2	− 4.2
Migration of tumor cells	5.4	− 2.4	− 3.8
Cell movement of phagocytes	5.4	− 1.1	− 3.4
Activation of cells	5.3	− 2.2	− 3.4
Cell movement of tumor cells	5.3	− 1.7	− 3.5
Activation of blood cells	5.3	− 1.8	0
Homing of cells	5.2	− 1.0	− 3.5
Cell movement of myeloid cells	5.2	− 0.8	− 3.4
Activation of leukocytes	5.2	− 1.7	− 3.0
Chemotaxis	5.2	− 0.9	− 3.4
Cell movement of leukocytes	5.1	− 0.7	− 3.3
Leukocyte migration	5.1	− 0.8	− 3.2
Activation of phagocytes	4.9	− 1.9	− 3.1
Activation of myeloid cells	4.7	− 1.4	− 3.1
Activation of mononuclear leukocytes	4.5	− 0.8	− 2.6
Migration of phagocytes	4.4	− 1.2	− 2.7
Quantity of cells	4.4	− 1.3	− 2.0
Chemotaxis of myeloid cells	4.4	− 0.8	− 2.7
Attraction of cells	4.4	− 0.8	− 3.1
Recruitment of cells	4.4	− 1.0	− 2.9
Expression of RNA	4.4	− 1.7	− 3.4
Transcription	4.4	− 1.3	− 3.0
Inflammatory response	4.3	− 1.5	− 3.4

Positive activation z-scores reflect the predicted activation of a disease or function (significant when > ~ 2)

Negative activation z-scores reflect the predicted suppression of a disease or function (significant when < ~− 2)

### BET proteins are necessary for induction of inflammation and adhesion mediator transcripts in monocytes

**Fig. 5:**
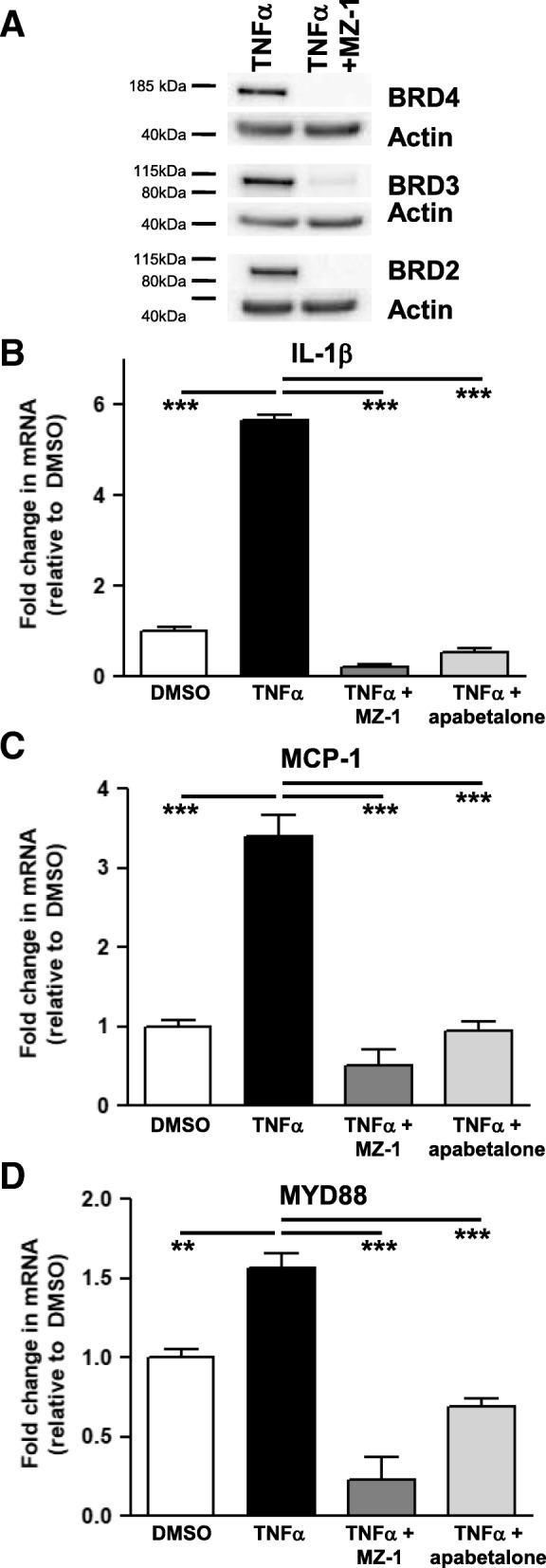
In THP-1 cells, MZ-1 and apabetalone prevent TNFα induction of key inflammatory and adhesion marker transcripts. **a** MZ-1 (6 h; 1 μM) degrades THP-1 BRD2, BRD3, and BRD4 as shown by western blot. **b**–**d** TNFα stimulation (2 h) fails to induce transcription of *IL-1β* (**b**), *MCP-1* (**c**), or *MYD88* (**d**) following MZ-1 pretreatment (6 h). Apabetalone (20 μM) pretreatment (6 h) decreases the transcripts of these genes. Statistical significance was determined through 1-way ANOVA analysis followed by Dunnett’s Multiple Comparison Test using TNFα response for the comparison, where *p < 0.05, **p < 0.01, ***p < 0.001

Confirmation of the central role of BET proteins in the expression of key inflammatory and adhesion molecules in monocytes was completed by treating THP-1 cells with MZ-1 and apabetalone and assessing subsequent gene expression changes. TNFα induction of monocyte genes is subtle compared to the response of endothelial cells. However, TNFα did upregulate THP-1 expression of several key VI mediators (Fig. 5; Table 6). This response is BET protein dependent; BET protein degradation (Fig. 5a) abolished the induction of *IL-1β* (Fig. 5b), monocyte chemoattractant protein (*MCP-1*) (Fig. 5c), and innate immune signal transduction adaptor (*MYD88*) (Fig. 5d). Apabetalone also suppressed the expression of these same monocyte genes (Fig. 5b–d) and the transcription of several other cytokine, chemokine, TLR signaling, and adhesion molecules (Table 6). Thus, BET proteins are required for the induction of pro-atherogenic and VI-related gene expression in both monocytes and endothelial cells, and targeted inhibition by apabetalone suppresses transcription in each cell type.

**Table 6:** THP-1 cytokine, chemokine, TLR signaling, and adhesion transcripts impacted by TNFα and apabetalone treatment

Function	Genes	TNFα		
		Fold induction	% reduction	
		Control	Apabetalone 5 μM	Apabetalone 20 μM
Cytokines	IL-1β	3.5	− 75	− 84
	TNFα	3.8	NS	− 54
Chemokines	CCR1	1.4	− 51	− 85
	CCR2	0.5	− 50	− 92
	MCP-1	3.7	− 77	− 91
TLR signaling	MYD88	2.6	− 39	− 71
	TLR4	0.7	NS	− 51
Adhesion molecules	CD44	1.8	− 26	− 39
	VLA-4	0.9	− 35	− 61

### Repression of transcription by apabetalone decreases the abundance of endothelial VI proteins

**Fig. 6:**
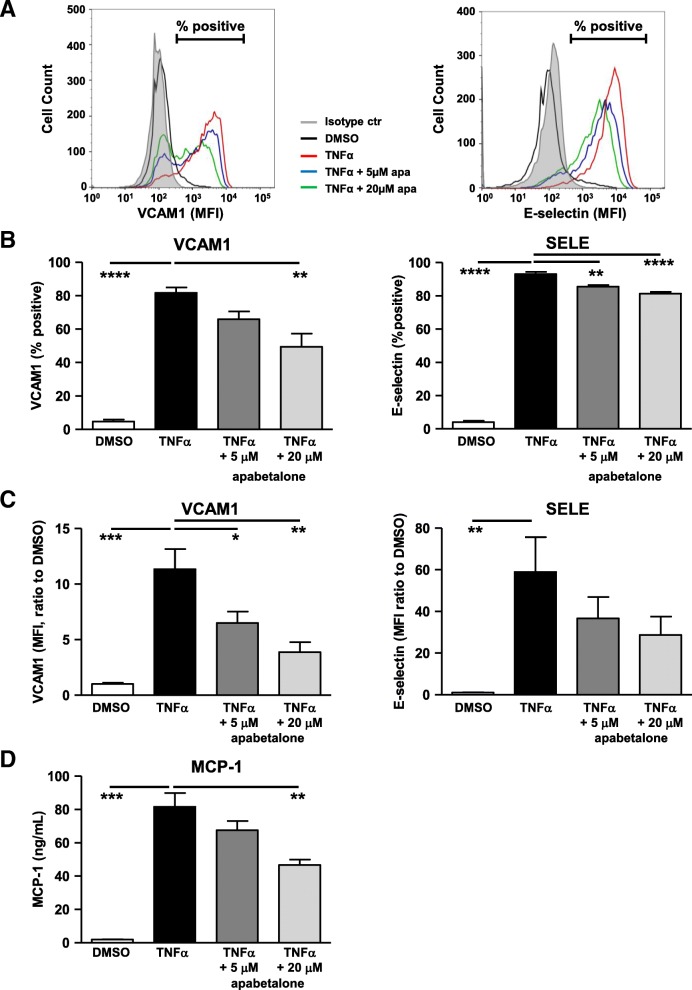
In HUVECs, apabetalone regulation of transcription reduces the abundance of VCAM-1 and MCP-1 proteins. HUVEC cells were stimulated with TNFα and co-treated with apabetalone for 4 h. The surface abundance of VCAM-1 (FITC-CD106) and SELE (APC-CD62E) were measured by flow cytometry. **a** Representative histogram overlay of HUVEC surface staining for VCAM-1 and SELE. Smaller peaks (% positive reduction) and left-ward curve shifts (MFI reduction) are both indications that there is a reduction in surface expression for the given protein. **b** Average of % positive cells expressing VCAM1 or SELE on the cell surface relative to the isotype control (the filled gray histogram as indicated in A). **c** Average mean fluorescent intensity (MFI) of VCAM1 and SELE on HUVEC surface relative to DMSO control. **d** HUVEC MCP-1 secretion is induced by overnight TNFα stimulation. Co-application with 20 μM apabetalone significantly reduces MCP-1 secretion (BD^TM^ cytometric bead array). In **b**–**d**, the results represent the mean of four independent experiments ± standard error. Statistical significance was determined through 1-way ANOVA analysis followed by Dunnett’s Multiple Comparison Test using TNFα response for the comparison, where *p < 0.05, **p < 0.01, ***p < 0.001

To determine if apabetalone’s transcriptional repression of VI mediators translated into a reduction in VI protein abundance, we quantified adhesion molecules on the surface of endothelial cells and chemokine release in response to treatment by flow cytometry. TNFα stimulation significantly increased the abundance of surface adhesion molecules (Fig. 6a, b, c) and evoked endothelial chemokine release (Fig. 6d). Apabetalone significantly reduced the percentage of HUVEC cells expressing vascular cell adhesion molecule 1 (VCAM-1) (Fig. 6a, b) and suppressed its surface abundance for more than 5 fold (Fig. 6c). SELE, an early adhesion marker for leukocyte recruitment, was also inhibited by apabetalone but to less extent than VCAM-1. Apabetalone resulted in a small but significant decrease of HUVEC expressing SELE proportional to the induced condition (Fig. 6b). The subtle change leads to a trending drop in SELE surface levels (Fig. 6c, *p* = 0.1). Endothelial secretion of MCP-1 was suppressed by apabetalone treatment (Fig. 6d). Overall, observations made at the gene transcript level largely translated into changes in protein expression.

### Monocyte adhesion to endothelial cells is suppressed by apabetalone

**Fig. 7:**
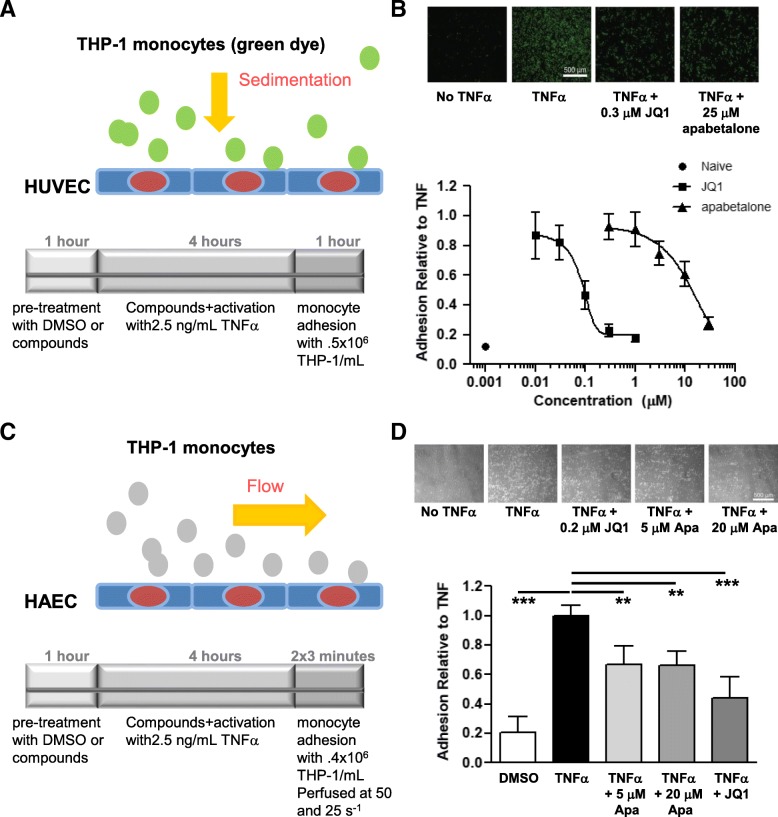
Apabetalone prevents monocyte adhesion to endothelial cells. **a** Static assay. Endothelial cell monolayer was pretreated with DMSO, JQ1, or apabetalone for 1 h before the addition of TNFα (2.5 ng/ml; 4 h incubation). Monocytes (0.5 × 10^6^cells/mL; loaded with calcein-AM) had 1 h to adhere to the monolayer before washes and fluorescence measures (plate reader). **b** Upper panel: fluorescent micrographs of monocyte adhesion to endothelial cells under static conditions. Lower panel: dose-response curves titrating JQ1 and apabetalone effect on monocyte /endothelial cell adhesion. JQ1 IC_50_ = 0.08 μM. apabetalone IC_50_ = 22 μM. **c** Flow assay. Pretreatment as in a; monocytes (0.4 × 10^6^ cells/ml) were perfused over the treated monolayer for 3 min with a flow rate of 50 s^−1^ then for another 3 min with a flow rate of 25 s^−1^. A high flow rate of 120 s^−1^ was applied to remove all unbound THP-1 cells, and images were acquired for analysis. **d** Upper panel: phase-contrast micrographs of monocyte adhesion to endothelial cells under flow conditions. Lower panel: apabetalone pretreatment prevents monocyte adhesion to endothelial cells under flow conditions; 0.2 μM JQ1 and 5 μM apabetalone had a comparable effect on adhesion. Statistical significance was determined through 1-way ANOVA analysis followed by Dunnett’s Multiple Comparison Test using TNFα response for the comparison, where *p < 0.05, **p < 0.01, ***p < 0.001

Monocyte adhesion to stimulated endothelial cells was quantified to investigate if apabetalone functionally alters adhesion under static or flow conditions. In both experiments, an endothelial monolayer was pretreated with BETi for 1 h before being additionally stimulated with TNFα for 4 h. In the static experiment, monocytes were allowed to sediment onto the endothelial monolayer for 1 h before non-adherent cells were removed and adhesion was quantified (Fig. 7a). BETi pretreatment of HUVECs resulted in the adherence of fewer monocytes (Fig. 7b). To increase the clinical relevance of the experiment, monocytes were perfused over a HAEC monolayer and adhesion was quantified (Fig. 7c). BETi pretreatment of HAECs also suppressed monocyte adhesion under flow (Fig. 7d). Remarkably, 0.2 μM JQ1 and 5 μM apabetalone, BETi with distinct chemical scaffolds and pan vs BD2 selectivity, had comparable effects, reducing adhesion by 35% (Fig. 7d). These data demonstrate that the BD2-selective BETi apabetalone does inhibit monocyte adhesion to endothelial cells, an early step in the atherogenic process.

### Apabetalone lowers the abundance of pro-atherogenic proteins in ASSURE CVD patient plasma

To determine the clinical effect of apabetalone on circulating protein abundance, SOMAscan™ 1.3K quantified the relative amount of ~ 1300 proteins in the plasma of ASSURE CVD patients treated with placebo (*n* = 47) or apabetalone (*n* = 47) for 26 weeks [27]. IPA® was used to predict upstream regulators, canonical pathways, and diseases and biological functions altered by apabetalone. Only proteins whose abundance changed by more than 10% with a *p* value less than 0.05 in apabetalone vs placebo-treated patients were included in this analysis. IPA® predicted inhibition of multiple pro-inflammatory upstream regulators by apabetalone treatment (Table 7, z-score <− 2), including macrophage migration inhibitory factor (MIF), TNFα, CD40 ligand (CD40LG), IFNγ, IL-6, granulocyte-macrophage colony stimulating factor (CSF-2/GM-CSF), and IL-1β. Interestingly, TNFα, IL-1β, IFNγ and IL-6 were also identified as upstream regulators in the endothelial gene expression data set analysis (Table 3). In plasma, apabetalone suppressed the abundance of many TNFα, IL-6, and IL-1β target proteins that correlate with CVD risk (Table 8). These include pro-inflammatory mediators and adhesion proteins that contribute to atherogenesis, and matrix metalloproteinases implicated in plaque instability. IPA® also predicted inhibition of the canonical “HMGB1 signaling” pathway by apabetalone in the analysis of both the patient plasma (Table 7) and endothelial cell data sets (Table 4). IPA® of the CVD patient plasma proteome further highlighted diseases and biological functions predicted to be inhibited by apabetalone (Table 9). The majority have strong immune cell activation, interaction, and recruitment components that are directly associated with VI and atherogenesis (Table 9). This data indicates that the BD2-selective BETi apabetalone lowers the abundance of VI mediators that play important roles in inflammation, vascular adhesion, and plaque stability in CVD patients.

**Table 7:** ASSURE patient plasma: IPA® analysis of the proteins in ASSURE patient plasma significantly affected by apabetalone versus placebo

Ingenuity® Pathway Analysis	Pathway/regulator	z-score	*p* value of overlap	Target molecules in dataset
*Canonical pathway*	HMGB1 signaling	− 1.7	2.8 × 10^−7^	TLR4, VCAM1, ICAM1, IL5, PTPN11, GRB2, OSM, IL17F, IL17B, TNFRSF11B
*Upstream regulators*	MIF	− 2.1	8.9 × 10^−10^	CD84, CRP, CXCL2, CXCL3, ICAM1, IL17RA, IL5, LTBR, MET, MMP3, TLR4, VCAM1
*Upstream regulators*	TNFα	− 2.1	1.8 × 10^−23^	ANGPT2, APCS, APP, ASGR1, BGN, BID, C5, CASP3, CCDC80, CCL1, CCL5, CD38, CHI3L1, CRP, CX3CL1, CXCL13, CXCL2, CXCL3, DLL4, ENTPD5, EPHB2, FCER2, FCGR2B, FLT4, FN1, FRZB, GFRA2, GSTP1, HSP90AB1, HSPA8, HSPD1, ICAM1, IGF1R, IL18BP, IL18R1, IL5, INSR, KIT, LTBR, LY96, LYN, MET, MMP12, MMP3, MST1, NME1, OSM, PAPPA, PDGFB, PI3, PLA2G2A, POSTN, PPIF, PRKCD, PTHLH, PTPN11, SERPIND1, SPARC, TEK, TF, TIE1, TLR4, TNFRSF11B, VCAM1
*Upstream regulators*	CD40LG	− 2.3	1.8 × 10^−4^	ANXA6, CCL5, CD38, CXCL2, FCER2, FCGR2B, ICAM1, JAK2, TNFRSF11B, TNFRSF17, VCAM1, ZAP70
*Upstream regulators*	IFNγ (complex)	− 2.4	1.8 × 10^−8^	CASP3, CCL5, CHI3L1, FN1, ICAM1, IL17RA, IL18BP, JAK2, LY96, TLR4
*Upstream regulators*	IL-6	− 2.5	1.1 × 10^−20^	ADGRE5, APCS, APP, BGN, CASP3, CCL3L1, CCL5, CD38, CRP, CXCL13, CXCL2, CXCL3, ENO2, FCER2, FN1, GRK2, HFE2, HPX, ICAM1, IL17F, IL5, IL6R, JAK2, KIT, LAG3, LY96, MET, MMP12, MMP3, PDGFB, PLA2G2A, PPBP, REG1A, ROR1, SST, TF, TLR4, TNFRSF11B, TNFRSF17, VCAM1
*Upstream regulators*	CSF2	− 2.6	1.6 × 10^−7^	ADGRE5, BID, BSG, CD33, CD38, CXCL2, FCGR2B, HSPD1, ICAM1, IL5, JAK2, LY96, MET, OSM, PPIF, RAD51, SLAMF7, TLR4
*Upstream regulators*	IL-1β	− 2.8	4.8 × 10^−19^	APCS, APP, ASAH2, BGN, C1R, CASP3, CCL1, CCL3L1, CCL5, CHI3L1, CRP, CX3CL1, CXCL13, CXCL2, CXCL3, DLL4, FCER2, FCGR2B, FN1, IBSP, ICAM1, IL17F, IL18R1, IL6R, INSR, LY96, MMP12, MMP3, OSM, PAPPA, PDGFB, PGAM1, PI3, PLA2G2A, POSTN, PRKCD, PTHLH, REN, SPARC, TLR4, TNFRSF11B, VCAM1

Negative activation z-scores reflect the predicted suppression of a pathway or an upstream regulator (significant when < ~− 2)

*P* value reflects the overlap between the genes in the data set that are significantly affected by treatment (> 10% change, *p* < 0.05) and the genes that are in the pathway (canonical) or regulated by the upstream regulator

Gene symbols are provided for the protein contributors that populate the identified pathways or upstream regulators

Italicized pathways and regulators are also found in the HUVEC NanoString IPA analysis (Table 4)

**Table 8:** ASSURE patient plasma: apabetalone reduces circulating TNFα targets that correlate with CVD risk in patient plasma

VI process	TNFα target	Protein symbol	Apabetalone versus placebo	
			% change	*p* value
Plaque stability	Stromelysin-1	MMP-3	− 26.8	0.005
Plaque stability	Macrophage metalloelastase	MMP-12	− 24.6	0.003
Inflammatory mediator Plaque stability	Fractalkine	CX3CL1	− 22.0	0.0003
Inflammatory mediator Plaque stability	C-reactive protein	CRP	− 21.3	0.02
Inflammatory mediator Plaque stability	Pappalysin-1	PAPPA	− 14.6	0.02
Inflammatory mediator Plaque stability	Osteoprotegerin	TNFRSF11B	− 14.0	0.003
Inflammatory mediator Plaque stability	Periostin	POSTN	− 13.3	0.01
Inflammatory mediator	Oncostatin-M	OSM	− 13.1	0.01
Atherogenesis Adhesion	Vascular cell adhesion protein 1	VCAM1	− 12.2	0.005
Inflammatory mediator	Toll-like receptor 4:Lymphocyte antigen 96 complex	TLR4 LY96	− 11.2	0.03
Inflammatory mediator	Serum amyloid P-component	APCS	− 10.8	0.001
Inflammatory mediator Plaque stability	Angiopoietin-2	ANGPT2	− 10.2	0.01

Compared to placebo treatment. Placebo group, *n* = 47; apabetalone treatment group, *n* = 47

**Table 9:** ASSURE patient plasma: IPA® diseases and biological functions identified in the analysis of the patient plasma proteome

Diseases and bio functions		
Ingenuity® Pathway Analysis	z-score	*p* value
*Response of granulocytes*	− 2.9	1.8 × 10^−13^
*Interaction of mononuclear leukocytes*	− 2.9	5.4 × 10^−13^
*Interaction of leukocytes*	− 2.8	3.7 × 10^−26^
*Binding of mononuclear leukocytes*	− 2.8	1.4 × 10^−12^
*Binding of leukocytes*	− 2.7	2.4 × 10^−25^
*Response of myeloid cells*	− 2.6	7.0 × 10^−15^
*Adhesion of mononuclear leukocytes*	− 2.6	7.8 × 10^−11^
*Response of myeloid leukocytes*	− 2.5	1.7 × 10^−14^
*Response of neutrophils*	− 2.5	5.9 × 10^−12^
*Adhesion of blood cells*	− 2.4	4.0 × 10^−23^
Outgrowth of cells	− 2.4	1.1 × 10^−10^
Neovascularization	− 2.4	2.5 × 10^−08^
*Interaction of lymphocytes*	− 2.4	2.0 × 10^−11^
*Interaction of blood cells*	− 2.4	4.7 × 10^−27^
Migration of tumor cell lines	− 2.4	1.5 × 10^−20^
Vascularization	− 2.3	1.4 × 10^−10^
Colony formation	− 2.3	9.7 × 10^−09^
*Binding of blood cells*	− 2.3	3.2 × 10^−26^
*Binding of lymphocytes*	− 2.3	4.6 × 10^−11^
*Adhesion of immune cells*	− 2.2	1.4 × 10^−23^
Cell death	− 2.2	3.4 × 10^−34^
Growth of neurites	− 2.2	7.6 × 10^−10^
Colony formation of cells	− 2.2	2.1 × 10^−08^

Positive activation z-scores reflect the predicted activation of a disease or function (significant when > ~ 2)

Negative activation z-scores reflect the predicted suppression of a disease or function (significant when < ~− 2)

*P* value reflects the overlap between the genes in the data set that are significantly affected by treatment (> 10% change, *p* < 0.05) and the genes that are in the diseases and function category

Italicized pathways have direct associations with vascular inflammation

## Discussion

In CVD and T2DM, elevated circulating cytokines potentiate VI through recruitment of leukocytes to the vascular endothelium. BET proteins are essential VI transcriptional regulators. Recent reports have demonstrated that pan-BETi and BRD2-4 siRNA prevent an increase in endothelial VI gene expression in response to inflammation [13, 28]. Here, we extend those findings, showing that MZ-1 degradation of BRD2-4 in both monocytes and endothelial cells suppresses TNFα induction of VI gene transcription.

Moreover, the BD2-selective BETi apabetalone has a similar impact on VI transcription as a pan-BETi: apabetalone treatment inhibits transcription by preventing BRD4 accumulation on VI gene enhancers and promoters. Transcriptional regulation by apabetalone critically reduces the abundance of surface adhesion molecules, impairing monocyte adhesion to stimulated endothelial cells. We also present clinical evidence that a BD2-selective BETi regulates circulating VI proteins. Analysis of the CVD patient plasma from the ASSURE phase II trial reveals that apabetalone treatment for 26 weeks reduces the abundance of multiple VI mediators. These mediators are involved in monocyte recruitment, adhesion, macrophage differentiation, and plaque stability, highlighting the clinical impact apabetalone has on VI.

Atherosclerosis contributes significantly to coronary artery disease, the leading cause of death in the developed world [29]. It is the combined consequence of chronic VI and lipid metabolism dysfunction, characterized by abnormally elevated levels of low-density lipoprotein (LDL) in the blood. Sub-endothelial retention of LDL, particularly oxLDL, is an initiating event in atherogenesis [30] and a contributing factor in plaque development [30]. However, combating rising LDL levels alone with HMG-CoA reductase inhibitors (statins) has failed to completely eliminate MACE events due to atherosclerosis [31]. Despite LDL management, approximately 1/3 of all CVD patients on statins still experience cardiovascular events due to residual inflammation [32]. Compounds that inhibit hyper-inflammatory signaling or block leukocyte-endothelial interactions are now being developed to combat residual inflammatory risk in atherosclerosis. For instance, direct suppression of inflammation with an interleukin IL-1β monoclonal antibody (canakinumab) reduced the relative risk of MACE by 15% in CVD patients over 3.7 years [33]. The Canakinumab Anti-Inflammatory Thrombosis Outcomes Study (CANTOS) has demonstrated that inflammation is another viable therapeutic target for the prevention of CVD-related events. The BD2-selective BETi apabetalone has also been shown to significantly reduce the relative risk of MACE. This may be attributed to its suppression of inflammatory gene expression and transcription of genes key to atherogenesis. These genes contribute to monocyte activation and recruitment, leukocyte capture, rolling, adhesion, firm adhesion, macrophage differentiation, plaque development, and stability (Fig. 1).

### Inflammation and monocyte recruitment

Apabetalone’s impact on inflammation is extensive, inhibiting the transcription of multiple chemokines/cytokines, cognate receptors, and components upstream of NF-κB activation (Fig. 2b). MCP-1, a chemokine secreted by both endothelial cells [34] and monocytes [35], is a key attractant in monocyte recruitment (Fig. 1). Previous studies have demonstrated that MCP-1 deficiency reduces atherosclerosis in ApoE (−/−) or LDLr (−/−) murine models [36] and that apabetalone inhibits TNFα-induced *MCP-1* gene expression in HAEC endothelial cells [23]. Here, we demonstrate that BET proteins are required for the induction of *MCP-1* transcription in endothelial cells and monocytes and that a BD2-specific BETi regulates *MCP-1* expression by displacing BRD4 from its promoter. Consequently, endothelial cells secrete less MCP-1. Apabetalone also reduces monocyte C-C motif chemokine receptor 2 (*CCR2*) expression, the cognate receptor of MCP-1. Through its reduction of both MCP-1 and CCR2, apabetalone treatment can be expected to reduce monocyte recruitment. Apabetalone also inhibits the transcription of major pro-inflammatory mediators that converge on NF-κB signaling through the TNFR, TLR, and IL-1R signaling pathways (Fig. 2). IPA® predicted that each of these pathways was regulated in apabetalone treated endothelial cells (NanoString) (Table 3). Functionally, apabetalone’s inhibition of these mediators is predicted by IPA® to inhibit immune cell activation, migration, and chemotaxis induced by TNFα stimulation (Table 5).

### Leukocyte capture

Leukocyte capture, the next step in atherogenesis, relies on endothelial cell surface expression of selectins (P-selectin and SELE) (Fig. 1) [25]. These adhesion molecules bind to leukocyte surface glycosylated ligands with high affinity in the presence of flow (shear stress) due to the “catch-bond” phenomenon [25, 37, 38]. Induction of *SELE* transcription not only requires BET proteins (Fig. 4c), it is the consequence of increased BRD4 occupancy on the *SELE* enhancer and promoter (Fig. 3e). Apabetalone inhibits *SELE* expression whether it is induced by TNFα, IL-1β, or LPS (Table 1). At the protein level, TNFα induction of SELE was variable, with apabetalone treatment resulting in a trending decrease in abundance (*p* = 0.1; Fig. 6b). These data indicate that apabetalone may impede early capture of monocytes by downregulating SELE expression.

### Slow leukocyte rolling and firm adhesion

Slow leukocyte rolling and firm adhesion follow initial capture and depend on leukocyte integrin (β2-integrin, Very Late Antigen-4 (VLA-4)) interactions with endothelial adhesion molecules (e.g., VCAM-1) (Fig. 1). VCAM-1 is only expressed in activated or dysfunctional endothelial cells in pro-atherogenic regions and areas in vessels where lesions are already established [39–41]. VCAM-1 interactions with monocyte VLA-4 are potentiated by “inside-out” and “outside-in” signaling, establishing firm adhesion and initiating the formation of docking structures [39, 42–44]. VCAM-1/VLA-4 binding also triggers the junction opening process, a requirement for trans-endothelial migration [25, 43, 45]. Previous studies have shown that treatment with anti-VCAM-1 antibody inhibits leukocyte adhesion, attenuates atherosclerosis, decreases plaque inflammation, and improves plaque stability in ApoE (−/−) mice [46]. In CVD patients, circulating levels of soluble VCAM-1 correlate positively with measures of carotid intima-media thickness and plaque destabilization [47, 48]. *VCAM-1* gene expression was inhibited by apabetalone treatment of endothelial cells in vitro and in ApoE (−/−) mice carotid arteries [23]. Here, we show that apabetalone regulates *VCAM-1* transcription through a BET-dependent mechanism in endothelial cells, and VCAM-1 surface abundance is substantially reduced as an outcome (Fig. 6a). In ASSURE CVD patient plasma, VCAM-1 protein abundance was also reduced by apabetalone treatment (12.2%, *p* = 0.005, vs placebo; Table 8). Firm adhesion is further compromised by apabetalone’s reduction of monocyte *VLA-4* transcripts that encode VCAM-1’s cognate receptor (Table 6). Thus, apabetalone is predicted to alter slow leukocyte rolling and firm adhesion by downregulating *VCAM-1* and *VLA-4* expression. Furthermore, here we show that in vitro, apabetalone does suppress monocyte adhesion to a TNFα stimulated endothelial monolayer under both static and flow conditions (Fig. 7), underscoring the functional consequence of BET-dependent inhibition on firm adhesion.

Firm adhesion also relies on interleukin 8 (IL-8) signaling [49]. IL-8 release from endothelial cells attracts leukocytes and neutrophils to sites of inflammation and infection and signals monocytes to increase their expression of the firm adhesion molecule β2 integrin [49]. High levels of IL-8 are detected in the serum of post-MI patients or patients with high cholesterol and unstable angina [50, 51]. IL-8 is also present in human atherosclerotic plaques, where it contributes to plaque instability by promoting matrix degradation [51]. IL-8 is thus a critical instigator of plaque rupture [51, 52]. Induction of *IL-8* transcription is BET-dependent. Apabetalone displaced BRD4 from the promoter of *IL-8*, where it accumulates following TNFα stimulation (Fig. 3e). Apabetalone’s epigenetic regulation of *IL-8* transcription likely contributes to decreases in firm adhesion and plaque rupture [22, 53].

### Macrophage differentiation, plaque development, and rupture

Firm adhesion enables monocyte transmigration through the endothelium. As monocytes arrive in the intima [25, 54], they differentiate into pro-inflammatory MI macrophages in response to cytokines and growth factors [25] (Fig. 1). CSF-2 (GM-CSF) is a prominent differentiation inducing cytokine/growth factor [55], whose expression is downregulated by apabetalone. IPA® predicted that CSF-2 signaling activity was inhibited in ASSURE CVD patient plasma by apabetalone (Table 7). This is expected to impede monocyte to M1 macrophage differentiation. In response to stimulation, macrophages are activated, which upregulates scavenger receptor expression. This enables the macrophages to internalize oxLDL and causes them to become lipid-laden foam cells (Fig. 1). Foam cells in the intima are a hallmark of early atherosclerosis [3, 54]. Through its regulation of inflammatory gene expression (*CSF-2*, *MCP-1*, *IL-1β*, C-X-C motif chemokine 10 (*CXCL10*), interleukin-15 (*IL-15*), and *IL-6* (Tables 1 and 2), apabetalone should interfere with the differentiation of monocytes to M1 macrophages to foam cells and, thus, plaque development.

Atherosclerotic plaque development is the combined consequence of chronic inflammation [56], environmental stresses (ROS, hypoxia), plaque structure (extracellular matrix, collagen, lipids), and evolving biological processes (infiltrating immune cells, apoptosis) [4]. Serious problems arise when the plaque ruptures, causing 67% of all fatal MIs and sudden cardiac deaths [4]. Matrix metalloproteinases (MMPs) are the modelers and remodelers of the plaque’s extracellular matrix, and as their abundance increases, the plaque’s stability is undermined [57]. TLR2 signaling directly induces MMP expression through MYD88 and NF-κB activation [58], while IL-8 indirectly regulates MMP abundance. The pan-BETi JQ1 decreased MMP expression and activity in HUVECs, mouse macrophages, and ECV304, an endothelial-like cell line [59]. BD2-selective apabetalone inhibits *IL-8*, *TLR2*, and endothelial cell expression of multiple plaque-destabilizing genes (Tables 1 and 2). The abundance of key plaque destabilizers (MMP-3, MMP-12, and pappalysin 1 (PAPPA) was substantially lower in the plasma of ASSURE CVD patients with apabetalone treatment (vs placebo, Table 8). Importantly, plaque vulnerability (plaque length, arc, and index) and MACE events also decreased with apabetalone treatment [22]. Animal studies had previously indicated that BETi affect plaque parameters; JQ1 attenuated angiotensin II-induced abdominal aortic aneurysm in ApoE (−/−) mice [59], and apabetalone reduced atherosclerosis in hyperlipidemic ApoE-deficient mice [23]. Thus, our data combined with previous studies strongly suggest that apabetalone may inhibit plaque development and promote plaque stability through its epigenetic regulation of transcription.

## Conclusions

Atherosclerosis is no longer defined solely as a lipid deposition disease. The CANTOS trial was the first phase III trial directly targeting inflammation as a CVD therapeutic strategy, and this novel approach resulted in MACE reduction [33]. Inflammation, however, is a critical form of cellular communication and is required to generate a defensive immune response. Blunted cytokine responses have the potential to increase the rate of infections and infestations; canakinumab did increase the incidence of fatal infections in patients by nearly two fold (0.31 vs. 0.18 per 100 person-years, *p* = 0.02) [33]. In contrast, apabetalone treatment, with its capacity to regulate multiple inflammatory factors (including cytokines, adhesion molecules, and APR proteins), does not impact the rate of infections or infestations in patients (safety data from phase II ASSURE, ASSERT, SUSTAIN, and ongoing phase III BETonMACE clinical trials) ([60, 61] data not shown). Apabetalone, therefore, is a CVD therapeutic candidate that effectively targets inflammation while maintaining a safety profile necessary for extended treatment.

Our in vitro work directly supports key findings of our clinical data: apabetalone, a BD2-selective BETi, downregulates the expression of multiple VI and atherogenic factors. Mechanistically, inflammation-driven gene expression requires direct BET protein interactions with chromatin and transcription factors, and apabetalone is capable of preventing BRD4 associations with key enhancers and promoters. Consequently, gene transcripts encoding proteins with roles in monocyte recruitment, leukocyte capture, rolling, adhesion, firm adhesion, macrophage differentiation, plaque development and stability are all downregulated by apabetalone (Fig. 1). The reduction in VI and atherogenic factors in CVD patient plasma correlates with the reduction in both MACE [53] and corresponding plaque parameters [22]. Apabetalone is at the front line of the new paradigm shift of targeting inflammation in atherosclerosis. Epigenetic inhibition of BET-dependent inflammatory gene expression by apabetalone is anticipated to contribute to the prevention of MACE in the ongoing study BETonMACE.

## Methods

### Cell culture

All cells were incubated at 37 °C in humidified atmosphere enriched with 5% CO_2_.

Pooled HUVECs (Lonza CC-2519, LOT 0000636877 and 0000475053) were expanded in Endothelial Cell Growth Base Media (R&D Systems CCM027) with Endothelial Growth Supplement, 1× Penicillin-Streptomycin (Gibco, Thermo Fisher), and 5ug/mL Plasmocin (invivo Gen). Cells were expanded until passage 3 or 4 (p3 or p4) only and immediately used in experiments.

HAECs (Lonza CC-2535, Lot 0000337673) were expanded in EGM-2 media (Lonza CC-3162) until passage 5, frozen (1 T75 per vial), and stored in liquid nitrogen. One vial was thawed into 4 T75 and expanded until 80% confluency (6–7 days).

THP-1 monocytes (Sigma or ATCC) were subcultured in RPMI-1640 media (Gibco/Life Technologies 11875-093) supplemented with 10% heat-inactivated FBS (Gibco 12483-020 Canada origin), 1× Penicillin-Streptomycin (Gibco, Thermo Fisher), 5ug/mL Plasmocin (invivo Gen), 1 mM sodium pyruvate (Gibco 11360-070), 10 mM HEPES pH 7.4 (Millipore TMS-003-C), and 0.05 mM beta-mercaptoethanol (Sigma M6250).

### Western blots

#### Nuclear translocation of RelA

HUVECs were treated with apabetalone (5 or 20 μM) or DMSO and TNFα (10 ng/ml) for 2 h. Nuclear and cytoplasmic lysates were prepared using NE-PER kit (ThermoScientific, 78833) with freshly added protease inhibitor cocktail (BioShop or Sigma-Aldrich), phosSTOP phosphatase inhibitor (Roche 04906837001), and 0.5uM TSA (HDAC inhibitor). Protein concentration was determined with BioRad DC assay and lysate was added to NuPAGE LDS sample buffer (Novex/Invitrogen/Life Technologies NP0007) and 20 ug of total protein was loaded onto a NuPAGE 4-12% Bis-Tris gel (Novex/Invitrogen/Life Technologies NP0321BOX). For immunoblotting, the following primary antibodies were used: anti-p65 (Abcam, ab16502), anti-phospho p65 S536 (Cell Signaling, 3033), anti-BRD2 (Bethyl, A302-583A), anti-alpha tubulin (Sigma, SAB3500023), and anti-β-actin conjugated to peroxidase (Sigma, A3854). Secondary antibodies used were goat anti-rabbit IgG H&L chain specific peroxidase (Calbiochem, 401353) and rabbit anti-chicken IgY H&L chain specific peroxidase (Abcam, ab6753). Immunoreactive proteins were visualized by the chemiluminescent reagent ECLTM prime (GE Healthcare, RPN2232).

#### PROTAC MZ-1 knock-down of BET proteins

HUVECs or THP-1 cells were pretreated with apabetalone (5 or 20 μM), MZ-1 (0.5 μM or 1 μM; Tocris #6154), or vehicle (DMSO) for 4 h. They were then stimulated with TNFα (10 ng/ml) in the presence of apabetalone, MZ-1, or DMSO for 2 h before lysates were harvested. mRNA was harvested using Catcher PLUS kits according to the manufacturer’s instructions (Life Technologies). Protein lysis buffer consisted of PBS (pH 7.4, Life Technologies) supplemented with 1% Nonidet® P40, 1% SDS, 0.5% sodium deoxycholate, 60 mM sodium fluoride, 1 mM sodium orthovanadate, 5 mM sodium pyrophosphate and freshly added protease inhibitor cocktail (BioShop or Sigma-Aldrich), phosSTOP phosphatase inhibitor (Roche 04906837001), and 0.5 μM trichostatin A (HDAC inhibitor). Lysates were sonicated at 75% output for 15 seconds with a Branson SLPt sonicator (Branson Ultrasonics, Danbury, CT). Insoluble material was removed by centrifugation at 10,000*g* at 4 °C for 3 min. The supernatant was removed and stored at − 80 °C until use. Protein concentration was determined with BioRad DC assay and lysate was added to NuPAGE LDS sample buffer (Novex/Invitrogen/Life Technologies NP0007) and 20 μg of total protein was loaded onto a NuPAGE 4-12% Bis-Tris gel (Novex/Invitrogen/Life Technologies NP0321BOX). For immunoblotting, the following primary antibodies were used: anti-BRD2 (Bethyl, A700-008), anti-BRD3 (Bethyl, A302-368A), anti-BRD4 (Bethyl, A700-005), anti-p65 (Abcam, ab16502), anti-phospho p65 S536 (Cell Signaling, 3033), and anti-β-actin conjugated to peroxidase (Sigma, A3854). Secondary antibody used was goat anti-rabbit IgG H&L chain specific peroxidase (Calbiochem, 401353). Immunoreactive proteins were visualized by the chemiluminescent reagent ECLTM prime (GE Healthcare, RPN2232).

### ChIP

Cell pellets (~ 20–30 million cells) were prepared from p3 HUVECs pretreated with apabetalone (5 or 20 μM) or vehicle (DMSO) for 1 h then stimulated with addition of TNFα (10 ng/ml) for 1 h. Cells were crosslinked with 1% formaldehyde, quenched with 125 mM Glycine, and washed pellets were shipped on dry ice to Active Motif (Carlsbad, CA) who prepared chromatin from the lysates, performed ChIP reactions and qPCR, and performed basic data analysis.

### Real-time PCR

mRNA was isolated from THP-1 cells and HUVEC cells, pretreated with apabetalone (5 or 20 uM) or vehicle (DMSO) for 1 h before stimulation with TNFα (10 ng/ml) in the presence of apabetalone or DMSO for 1 h (Additional file 1: Figure S1) or 4 h (all other experiments) (Catcher PLUS kits; Life Technologies). Taqman PCR assays were obtained from Applied Biosystems/Life Technologies. Real-time PCR was used to determine the abundance of the transcript relative to the endogenous control cyclophilin in the same sample using the RNA Ultrasense One-step qRT-PCR kit (Life Technologies). Data was acquired using a ViiA-7 Real-Time PCR apparatus (Applied Biosystems). Analysis was performed as 2^ (CT cyclophilin – CT marker) and results were normalized to DMSO treated samples.

### NanoString

HUVECs were pretreated with apabetalone (5 or 20 μM) or vehicle (DMSO) for 1 h before addition of TNFα (10 ng/ml) for 4 h. HUVEC total RNA was then isolated (RNEasy isolation kit, Qiagen) and sent to NanoString for multiplex gene expression analysis (255 human genes) with the nCounter® Inflammation v2 Panel. NanoString was run by the University of Alberta pathology core and data was analyzed in house with the nSolver^TM^ software and Ingenuity^R^ Pathway Analysis software (Oct 2018 update). Genes with fold change > 1.3 and <− 1.3 were used in Ingenuity^R^ Pathway Analysis.

### Flow cytometric analysis of VCAM-1 and SELE HUVEC surface expression and MCP-1 secretion

HUVECs were stimulated for 4 h with DMSO or TNFα ± apabetalone (5 or 20 μM). Surface expression of VCAM-1 and SELE was labeled with FITC conjugated anti-CD106 and APC conjugated anti-CD62E (BD Bioscience) and quantified by flow cytometry. Mean fluorescence intensity (MFI) relative to the unstimulated DMSO control were reported. HUVECs were stimulated for overnight with DMSO or TNFα ± apabetalone (5 or 20 μM). Supernatant was collected and MCP-1 secretion was measured by a cytometric bead array (MCP-1 flex set, BD Bioscience), and detected by flow cytometry.

### Static adhesion assay

HUVECs were seeded into black/clear bottom 96-well plates (Thermo Scientific™ Nunc™ MicroWell™ 96-Well Optical-Bottom Plates with Polymer Base, Thermo Scientific cat #165305) at a density of 12,500 cells/well. Twenty-four hours after seeding, cells were treated as dep. An initial 1-h pre-conditioning was followed by 4 h 2.5 ng/mL TNFα (Peprotech cat #300-01A-50UG, Lot 0607B25 G2516) treatment (in presence of compound or DMSO). THP-1 cells were stained with 5 μM calcein-AM (Thermo Fisher Scientific cat # C3100MP) in serum-free RPMI-1640 media at a density of 2.5 × 10^6^ cells/ml for 30 min at 37 °C, 5% CO_2_ and washed twice in THP-1 media. Endothelial cells were washed once in THP-1 media before addition of 200 μl/well of calcein-AM labeled THP-1 at a density of 0.5 × 10^6^ cells/ml for 1 h at 37 °C and 5% CO_2_. Unbound monocytes were removed by 4–6 washes (90° turn of plate between wash) with pre-warmed THP-1 media. Once all unbound THP-1 cells were removed, plates were washed 2× with PBS, fixed in 4% paraformaldehyde for 5 min, and washed 2× with PBS. The amount of bound monocytes was indirectly determined by measuring the fluorescent signal on a plate reader (SpectraMax M2, Molecular Devices). A 9-point well scan was performed at an excitation of 485 nm and emission of 520 nm. Additionally, representative images were captured on an Eclipse TS100 microscope (Nikon) with a × 10 objective and a Zyla sCMOS camera (Andor).

### Flow adhesion assay

HAECs were seeded onto gelatin-coated standard glass microscope slides at a density of 0.2 × 10^6^ cells/slide and treated according to the outlined regimen (Fig. 5b). Following treatment, HAECs were mounted in the flow chamber and placed on an inverted Eclipse TS100 microscope (Nikon) with a × 10 objective and a Zyla sCMOS camera (Andor). A heat lamp maintained the temperature at approximately 37 °C. Flow chambers are composed of a 0.01-in.-thick silicone rubber gaskets with a removed rectangular section to form the flow channel (0.5 × 1.97 in.) and a plexiglass top plate providing the inlet and outlet as described in Viegas ([62]). THP-1 cells were perfused through the flow chamber in full THP-1 media at a density of 0.4 × 10^6^ cells/ml using a 10 cc syringe mounted on a syringe pump (KDS 200, dual syringe infusion pump, KD Scientific) according to the following scheme:

• 3 min at 50 s^−1^

• 3 min at 25 s^−1^

• 120 s^−1^ to take pictures

A 2-min video was captured at 50 and 25 s^−1^ shear rate and 10 images taken at a high shear rate of 120 s^−1^ where all unbound THP-1 are removed. Monocyte adhesion was quantified in ImageJ using the “Find Maxima” function.

### SOMAScan^TM^ Proteomic analysis

The ASSURE study (NCT01067820) design and rationale has been published previously [63]. Briefly, it was a phase II, placebo-controlled, multi-center, double-blind study. The study was conducted in accordance with the ethical standards of the responsible committee on human experimentation (institutional and national) and with the Helsinki Declaration of 1975, as revised in 2000. Informed consent was obtained from all study participants. Participants all had unstable CAD, with at least one > 20% lumen stenosis in a native epicardial coronary artery on visual estimation of a clinically indicated coronary angiogram. Patients received placebo or 100 mg apabetalone twice per day. All patients also received standard medical therapy, either atorvastatin (10, 20, or 40 mg) or rosuvastatin (5, 10, or 20 mg). Baseline and end-of-study (26 weeks) plasma samples (47 placebo and 47 apabetalone treated) from ASSURE were analyzed using the SOMAScan^TM^ 1.3 K proteomic technology (Somalogic Inc., Boulder, CO). The abundance of 1305 proteins was assessed. Wilcoxon signed-rank tests were run versus baseline. Mann-Whitney *U* test were run to compare median change and percent change between apabetalone treated patients and placebo. IPA® software analyzed changes (> 10%, *p* < 0.05) in proteins affected by apabetalone treatment (versus placebo) and predicted its impact on canonical pathways, upstream regulators, and diseases and biological functions.

## Additional files

Additional file 1: **Figure S1.** Overall abundance of BRD4 protein levels in HUVECs did not change with apabetalone treatment (western blot). HUVEC cells were co-treated with TNFα and either apabetalone, RVX compound B, or MZ-1 (0.2uM) for 24hrs. Western blot of protein lysates was probed with anti-BRD4 antibody (Bethyl, A700-005) and goat anti-rabbit IgG H&L chain specific peroxidase (Calbiochem, 401353). Anti-β actin conjugated to peroxidase (Sigma, A3854) was used as a loading control. **Figure S2.** In HUVECs, 1 hour apabetalone pretreatment significantly inhibited TNFα-induced expression of MCP-1, SELE, and VCAM-1 (1 hour stimulation). Cells treated in parallel were processed for ChIP or RT-PCR according to the protocols found in Methods. Statistical significance was determined through 1-way ANOVA analysis followed by Tukey's Multiple Comparison Test, where ***p<0.001 (PPTX 203 kb)

## Data Availability

The datasets generated and/or analyzed during the current study are not publicly available. Reasonable requests for data will be considered. All genes, proteins, pathways, upstream regulators, and diseases and biological functions discussed are provided in the Tables within the paper itself.

## Abbreviations

ApoE (−/−): Apolipoprotein E-deficient
APC: Allophycocyanin
APCS: Serum amyloid P-component
ANGPT2: Angiopoietin-2
ASSURE: ApoA-I Synthesis Stimulation and Intravascular Ultrasound for Coronary Atheroma Regression Evaluation
BET: Bromodomain and extraterminal protein
BETi: Bromodomain and extraterminal protein inhibitor
BD1: Bromodomain 1
BD2: Bromodomain 2
BRD2: Bromodomain containing 2
BRD3: Bromodomain containing 3
BRD4: Bromodomain containing 4
C1s: Complement C1s
CANTOS: Canakinumab Anti-Inflammatory Thrombosis Outcomes Study
CCR1: C-C motif chemokine receptor 1
CCR2: C-C motif chemokine receptor 2
CD106: Cluster of differentiation 106 (VCAM-1)
CD40LG: Cluster of differentiation 40 ligand
CD44: Cluster of differentiation 44
CD62E: Cluster of differentiation 62E (SELE)
CFB: Complement factor b
ChIP: Chromatin immunoprecipitation
CO_2_: Carbon dioxide
COX-2: Cyclooxygenase 2
CRP: C-reactive protein
CSF-2/GM-CSF: Granulocyte-macrophage colony stimulating factor
CVD: Cardiovascular disease
CX3CL1: C-X3-C motif chemokine ligand 1
CXCL10: C-X-C motif chemokine 10
CXCL3: C-X-C motif chemokine ligand 3
DMSO: Dimethyl sulfoxide
EGM-2: Endothelial cell growth medium-2
FBS: Fetal bovine serum
FITC: Fluorescein isothiocyanate
HAEC: Human aortic endothelial cell
HEPES: 4-(2-hydroxyethyl)-1-piperazineethanesulfonic acid
HDAC: Histone deacetylase
HGF: Hepatocyte growth factor
HMGB1: High mobility group box 1
HMG-CoA: 3-Hydroxy-3-Methylglutaryl-Coenzyme A
HUVEC: Human umbilical vein endothelial cell
IgG: Immunoglobulin G
IL-1a: Interleukin 1 alpha
IL-1b: Interleukin 1 beta
IL-1R: Interleukin 1 receptor
IL-15: Interleukin 15
IL-18: Interleukin 18
IL-6: Interleukin 6
IL-8: Interleukin 8
IFIT2: Interferon-induced protein with tetratricopeptide repeats 2
IFNg: Interferon gamma
IPA: Ingenuity pathway analysis
IRF-1: Interferon regulatory factor 1
JNK: Jun N-terminal kinase
LDL: Low-density lipoprotein
LDLR (−/−): LDL receptor-deficient
LPS: Lipopolysaccharide
LTB: Lymphotoxin beta
MACE: Major adverse cardiac events
MCP-1: Monocyte chemoattractant protein 1
MI: Myocardial infarction
MIF: Macrophage inhibitory factor
MMP: Matrix metalloproteinase
MMP3: Matrix metalloproteinase 3
MMP12: Matrix metalloproteinase 12
mRNA: Messenger ribonucleic acid
MYD88: Innate immune signal transduction adaptor
NF-kB: Nuclear factor kappa-light-chain-enhancer of activated B cells
OPG: Osteoprotegerin
OSM: Oncostatin M
oxLDL: Oxidized LDL
PAPPA: Pappalysin-1
PCR: Polymerase chain reaction
PBS: Phosphate-buffered saline
POSTN: Periostin
PROTAC: Proteolysis targeting chimeric molecule
P-TEFb: Positive transcription elongation factor
RELA: RELA proto-oncogene, NF-KB Subunit
RELB: RELB proto-oncogene, NF-KB Subunit
RNA: Ribonucleic acid
ROS: Reactive oxygen species
RPMI-1640: Roswell Park Memorial Institute-1640
RR: Relative risk
SAPK: Stress-activated protein kinase
SE: Super-enhancer
SELE: E-selectin
T2DM: Type 2 diabetes mellitus
TGFB3: Transforming growth factor beta 3
TICAM1: Toll-like receptor adaptor molecule 1
TLR: Toll-like receptor
TLR2: Toll-like receptor 2
TLR3: Toll-like receptor 3
TLR4: Toll-like receptor 4
TLR4 LY96: Toll-like receptor 4:lymphocyte antigen 96 complex
TNFAIP3: TNF alpha-induced protein 3
TNFa: Tumor necrosis factor alpha
TNFR: Tumor necrosis factor receptor
TNFRSF11B: TNF receptor superfamily member 11b
TRADD: TNFRSF1A associated via death domain
TREM1: Triggering receptor expressed on myeloid cells 1
TSA: Trichostatin A
VCAM-1: Vascular cell adhesion molecule 1
VI: Vascular inflammation
VLA-4: Very late antigen-4
