# Supplementary material for: Apabetalone (RVX-208) reduces vascular inflammation in vitro and in CVD patients by a BET-dependent epigenetic mechanism
Source: Clin Epigenetics. 2019 Jul 12;11:102. doi: 10.1186/s13148-019-0696-z (PMC6626370; doi:10.1186/s13148-019-0696-z)
Supplement: Supplementary file 1 — Figure S1. Overall abundance of BRD4 protein levels in HUVECs did not change with apabetalone treatment (western blot). HUVEC cells were co-treated with TNFα and either apabetalone, RVX compound B, or MZ-1 (0.2uM) for 24hrs. Western blot of protein lysates was probed with anti-BRD4 antibody (Bethyl, A700-005) and goat anti-rabbit IgG H&L chain specific peroxidase (Calbiochem, 401353). Anti-β actin conjugated to peroxidase (Sigma, A3854) was used as a loading control. Figure S2. In HUVECs, 1 hour apabetalone pretreatment significantly inhibited TNFα-induced expression of MCP-1, SELE, and VCAM-1 (1 hour stimulation). Cells treated in parallel were processed for ChIP or RT-PCR according to the protocols found in Methods. Statistical significance was determined through 1-way ANOVA analysis followed by Tukey's Multiple Comparison Test, where ***p<0.001 (PPTX 203 kb) [file 13148_2019_696_MOESM1_ESM.pptx]

## Slide 1
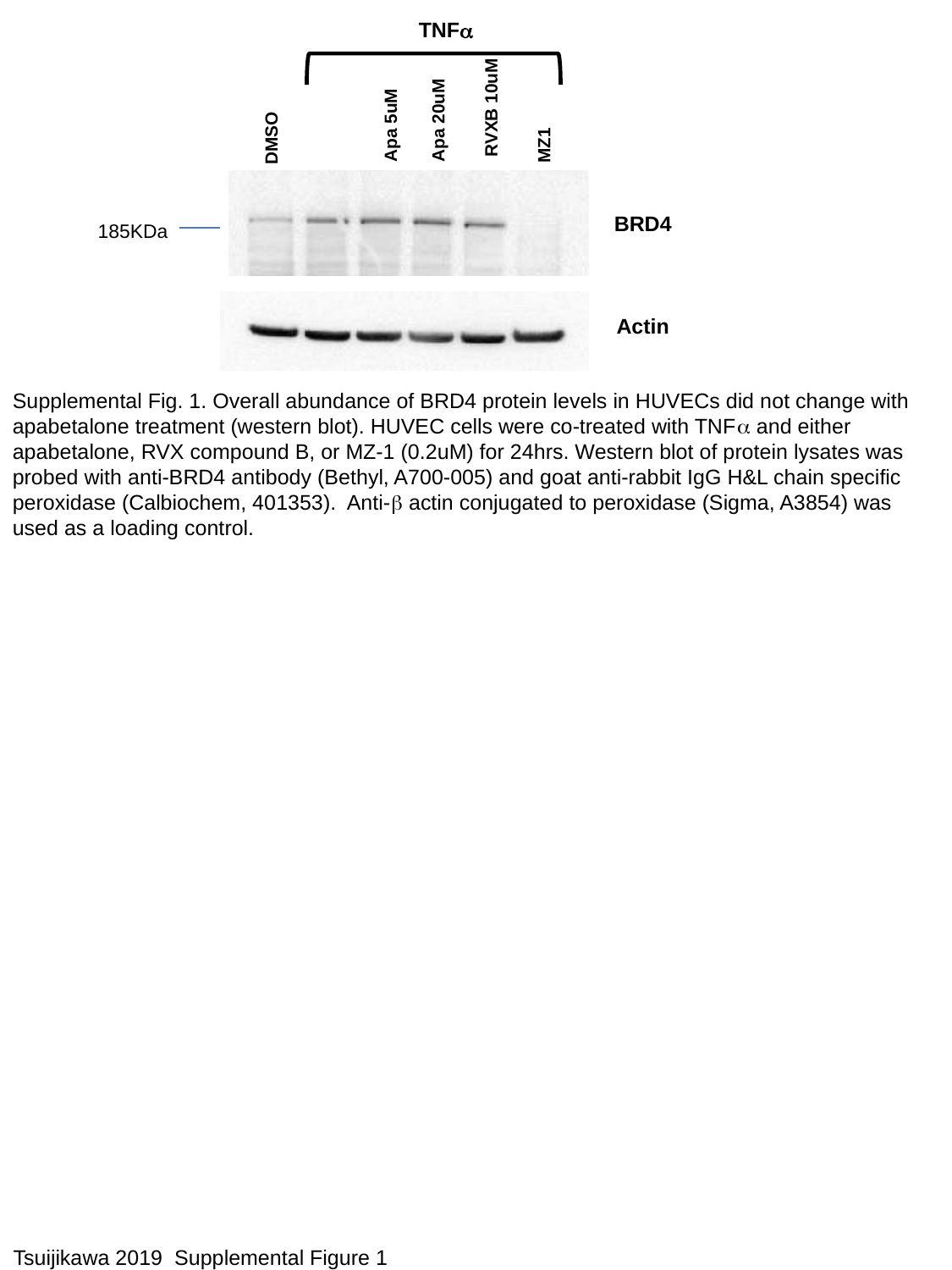

TNFα
 RVXB 10uM
Apa 20uM
Apa 5uM
DMSO
MZ1
BRD4
185KDa
Actin
Supplemental Fig. 1. Overall abundance of BRD4 protein levels in HUVECs did not change with apabetalone treatment (western blot). HUVEC cells were co-treated with TNFα and either apabetalone, RVX compound B, or MZ-1 (0.2uM) for 24hrs. Western blot of protein lysates was probed with anti-BRD4 antibody (Bethyl, A700-005) and goat anti-rabbit IgG H&L chain specific peroxidase (Calbiochem, 401353). Anti-β actin conjugated to peroxidase (Sigma, A3854) was used as a loading control.
Tsuijikawa 2019 Supplemental Figure 1

## Slide 2
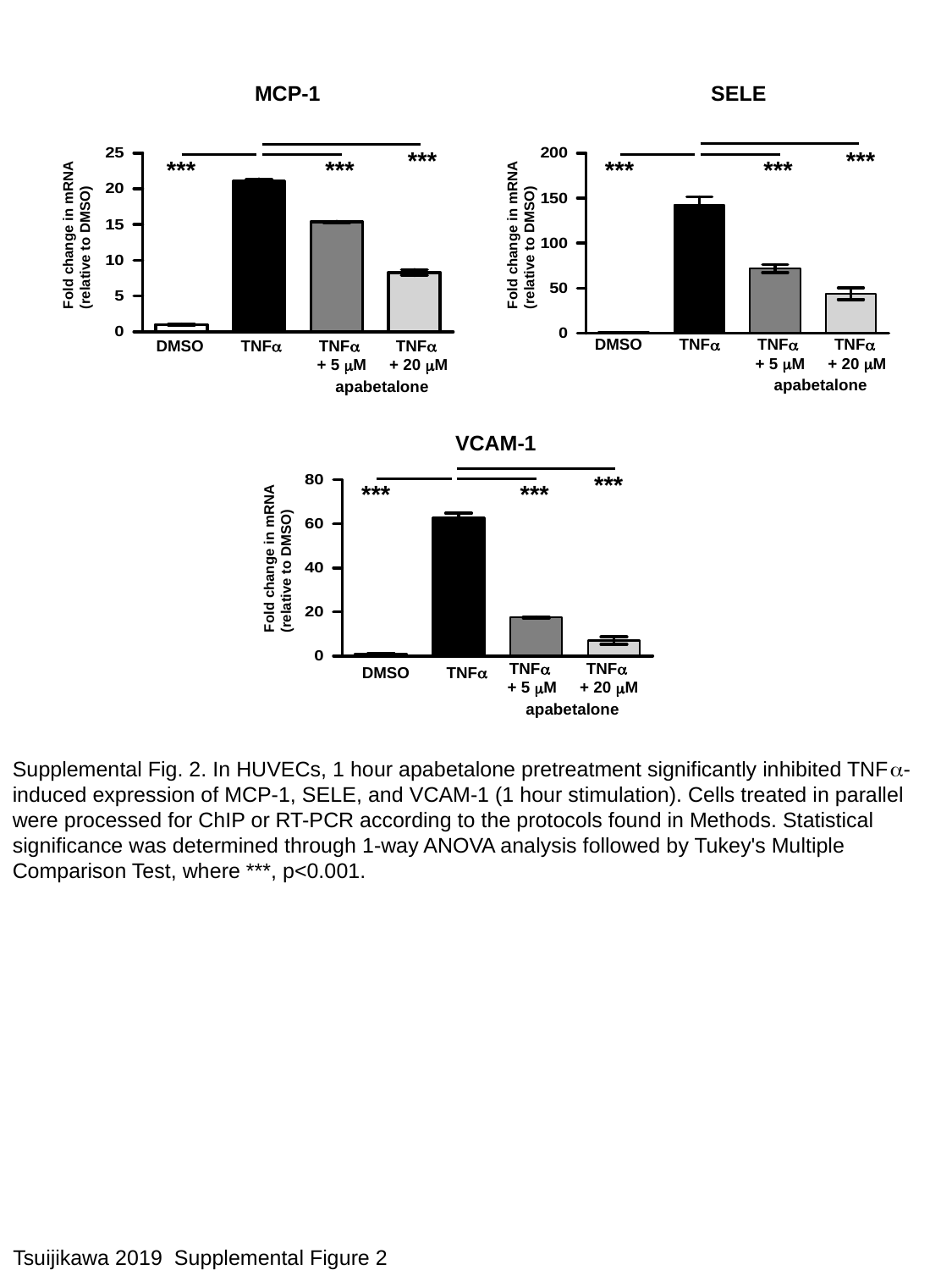

MCP-1
SELE
***
***
***
***
***
***
Fold change in mRNA
(relative to DMSO)
Fold change in mRNA
(relative to DMSO)
DMSO
TNFα
TNFα
+ 5 μM
TNFα
+ 20 μM
DMSO
TNFα
TNFα
+ 5 μM
TNFα
+ 20 μM
apabetalone
apabetalone
VCAM-1
***
***
***
Fold change in mRNA
(relative to DMSO)
TNFα
+ 5 μM
TNFα
+ 20 μM
DMSO
TNFα
apabetalone
Supplemental Fig. 2. In HUVECs, 1 hour apabetalone pretreatment significantly inhibited TNFα-induced expression of MCP-1, SELE, and VCAM-1 (1 hour stimulation). Cells treated in parallel were processed for ChIP or RT-PCR according to the protocols found in Methods. Statistical significance was determined through 1-way ANOVA analysis followed by Tukey's Multiple Comparison Test, where ***, p<0.001.
Tsuijikawa 2019 Supplemental Figure 2
